# Modeling Differential Effects of Maternal Dietary Patterns across Severity Levels of Preterm Birth Using a Partial Proportional Odds Model

**DOI:** 10.1038/s41598-020-62447-4

**Published:** 2020-03-26

**Authors:** Aweke A. Mitku, Temesgen Zewotir, Delia North, Prakash Jeena, Rajen N. Naidoo

**Affiliations:** 1 School of Mathematics, Statistics and Computer Science, College of Agriculture Engineering and Science, University of KwaZulu-Natal, Durban, South Africa; 2 Discipline of Pediatrics and Child Health, School of Clinical Medicine, College of Health Sciences, University of KwaZulu-Natal, Durban, South Africa; 3 Discipline of Occupational and Environmental Health, School of Nursing and Public Health, College of Health Sciences, University of KwaZulu-Natal, Durban, South Africa; 4 Department of Statistics, Bahir Dar University, Bahir, Dar Ethiopia

**Keywords:** Risk factors, Mathematics and computing

## Abstract

Preterm birth is a common cause of death worldwide of children under the age of five years. This condition is linked with short and long term neonatal morbidity and mortality. Maternal nutrition during pregnancy has a profound effect on fetal growth and development and subsequently also on the incidence of preterm birth. The aim of this study was to assess the differential effect of dietary patterns of pregnant women across ordered levels of preterm birth. Dietary assessments were performed using a food frequency questionnaire, presented to 687 pregnant women, in the “Mother and Child in the Environment” birth cohort during the period of 2013 to 2017. Each pregnancy resulted in a live birth. Eight dietary patterns were extracted, using exploratory factor analysis. The partial proportional odds model was employed to model severity levels of preterm birth. The partial proportional odds model has been recognized to be a flexible approach since it allows the effect of predictor variables to vary across categories of the ordinal response variable of interest. Women with increased consumption of vegetable-rich foods showed a reduced risk of very to moderately preterm birth incidence (AOR = 0.73, 95% CI = (0.531, 0.981), p = 0.036). Lower odds of very/moderately preterm birth compared to late preterm or term birth were observed for women following “nuts and rice foods” dietary pattern (AOR = 0.25, 95% CI = (0.099, 0.621), p = 0.003). High dietary consumption of starch foods dietary pattern (AOR = 2.09, 95% CI = (1.158, 3.769), p = 0.014) was associated with the most severe level of preterm birth outcome incidence, i.e. very/moderately preterm birth. The partial proportional odds modeling allowed the description of the effect of maternal dietary patterns across the different severity levels of preterm birth.

## Introduction

The World Health Organization (WHO) has defined preterm birth as the spontaneous or induced live delivery of babies before 37 completed weeks of gestation^1^. Preterm birth is associated with short and long term neonatal morbidity and mortality^2–5^ and is the second-leading cause of under-five mortality, worldwide^1^. The global prevalence of preterm birth is 10%, accounting for 15 million births, globally and approximately 50% of all perinatal deaths every year^1,6^. In South Africa, more than 8 out of 100 babies are preterm. The country was ranked 24^th^ out of 184 countries in 2010, for the number of newborn deaths, due to complications from preterm birth^1^.

With a few exceptions, the rate at which preterm birth occurs has grown in both developed and developing countries, over the last decade^1,5^. Increased maternal age during pregnancy, infertility treatment and maternal health conditions are the leading causes (1). However, changes in obstetric practice (evidenced by an increase in induced deliveries and cesarean section, are possible additional reasons for the increased incidence of preterm births^1^. The highest increase in preterm birth incidence was observed to be in the moderate preterm category (32–33 weeks’ gestation), as well as the late preterm category (34–36 weeks’ gestation)^7,8^. Although considerable attention has been paid to preterm birth as a whole, outcomes vary with the subcategories of preterm birth^9–11^. The rate of preterm birth has increased by 33% in the last 25 years, almost entirely due to the rise in late preterm births^12^. The extent of the increased risk of preterm birth associated with socio-economic disadvantage and other risk factors generally increased with an increasing severity level of preterm birth^11^.

Maternal nutrition has an effect on fetal growth^13–16^ and preterm deliveries^17–21^. Several studies have yielded varying results concerning the associations between single foods or nutrients during pregnancy and preterm birth incidence^22–26^. Other studies looking at dietary patterns of foods in combination during pregnancy have allowed for the interactive effect between nutrients and timing of deliveries to be studied^27,28^. As an example, a western diet, which is described as consisting of a high consumption of fried and processed meats, is associated with an increased risk of preterm birth^24,29^, while prudent diets, which are rich in vegetables and fruits, have been associated with a lower occurrence of preterm birth^23^.

The complexity of preterm birth suggests that a simple binary logistic regression approach fails to identify exposure effects at different severity levels of preterm birth^24,29–31^. Many researchers have applied newer statistical and scientific methods to identify the effect of dietary patterns on the incidence of preterm birth. Since it is not appropriate to assume that all the factors have the same effect across different severity levels of preterm birth, an ordinal logistic regression model was proposed for such investigation by Walker and Duncan^32^ and was later referred to as the proportional odds model^33–36^. The proportional odds model is a generalization of a binary logistic regression model, in which the response variable has more than two ordinal categories. The use of ordinal polytomous responses has increased significantly in health science studies on quality of life, defining health status indicators, the severity of certain diseases and the effectiveness of post-operative procedures^36^. Data in such studies are commonly evaluated by the proportional odds model^33–35,37^. The partial proportional odds model combines the ordered arrangement in ordinal models, while allowing for particular independent variables to affect different levels of the outcome variables^28^.

Even though maternal diet has been considered as a determinant of preterm birth prevalence in previous studies, the association with preterm birth is not well established in South Africa. South Africa is experiencing a transition in dietary patterns, from that of traditional diets to a more “Western” diet, high in fats and sugar^38^. It is, therefore, useful to assess the association of dietary patterns during pregnancy with preterm birth, in order to make informed decisions on dietary interventions during pregnancy^27^. We used a novel modeling strategy, the partial proportional odds model, which allows for more nuanced insights on the effect of dietary patterns across different severity levels of preterm birth than other approaches, such as the binary and multinomial logistic regression models. This paper attempts to model the differential effect of maternal dietary patterns, across the severity levels of preterm birth using the partial proportional odds model.

## Methods

### Data

The Mother and Child in the Environment (MACE) birth cohort is based among low-income communities in Durban, South Africa. The study enrolled 996 pregnant women between March 2013 and May 2017. Participants in the study were selected from public sector antenatal clinics in the industry dense residential areas in south Durban (Merebank, Bluff, Wentworth, and Austerville), as well as from residential located areas in the less heavily industrial areas in the north of Durban (Kwa Mashu, Newlands and Inanda). All pregnant women that met inclusion and exclusion criteria, were enrolled in the study and followed up during their pregnancy, through to labour and delivery. The inclusion criteria included gestational age less than 20 weeks and resident for the full duration of the pregnancy in the geographical area within which the clinic was located. Women with multiple pregnancies were excluded. Ethical approval was obtained from the University of KwaZulu-Natal’s Biomedical Research Ethics Committee, and each participant provided informed consent, participation was voluntary and withdrawl from the study at any point was allowed.

Among the 996 enrolled pregnant women, 309 subjects were excluded due to miscarriages, termination of pregnancy and loss to follow up. The study analysed data on 687 pregnant women in the cohort. The food frequency questionnaire was administered to the mothers in the third trimester. Dietary patterns were based on the 75 food items commonly used in the maternal diet. According to WHO^1^ and Donoghue *et al*.^39^, preterm birth is classified as very preterm (<32 weeks), moderately preterm (32–33 weeks), late preterm (34–37 weeks), and term (38–42 weeks).

### Data reduction

**Figure 1:**
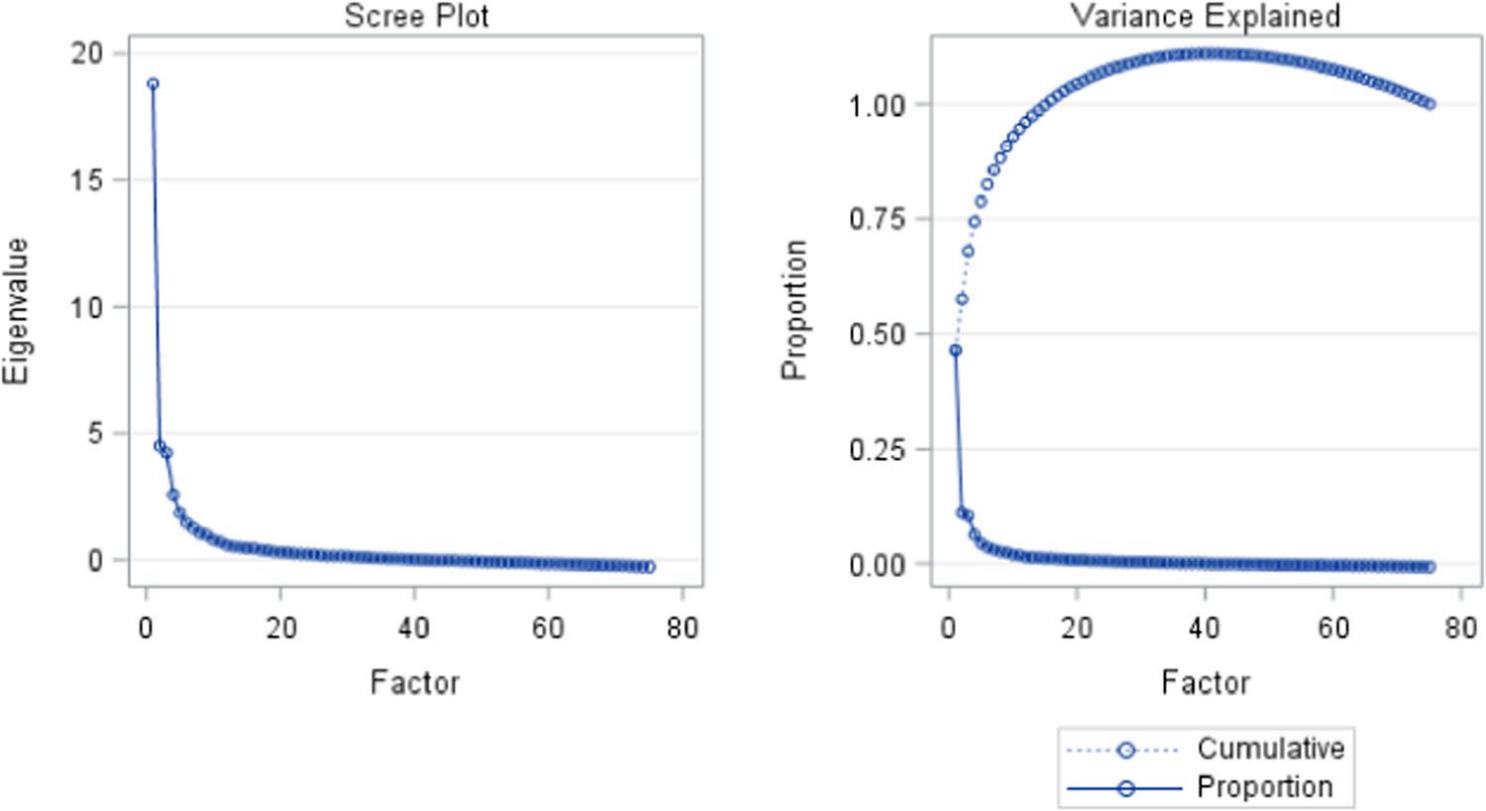
Scree plot and percentage of variance explained by factor analysis on maternal dietary patterns.

To reduce the 75 dietary variables into a set of manageable latent characteristics, with minimal loss of information, exploratory factor analyses with a Promax rotation method was used. A scree plot (Fig. 1), along with the percentage of variance explained by each factor, was used to determine the number of latent factors. Accordingly, eight latent dietary factors that explain dietary patterns were identified. Collectively these factors explained 88.3% of the variability within the sample. These factors were labeled as ‘energy foods and snacks’, ‘spreads and fast foods’, ‘butter, junk foods, and juices’, ‘protein-rich foods’, ‘starch foods’, ‘nuts and rice foods’, ‘vegetable-rich foods’ and ‘alcoholic drinks’. The summary of results, with the factor loadings and naming of the dietary patterns, is given in Table 1.

**Table 1:** Factor loadings of different food items on the eight latent dietary factors identified using factor analysis with Promax rotation.

Dietary patterns	Food items	Factor loadings coefficient*	Cumulative variance explained (%)
“Energy foods and snacks”	Energy bars	0.87486	46.41%
	Energy drinks	0.85251	
	Ice cream	0.76687	
	Chocolate	0.74735	
	Drinking yogurt	0.70387	
	Milk drinks	0.70378	
	Milkshake	0.66289	
	Fruit salad	0.63208	
	Cheese sauce	0.62704	
	Cheese	0.56129	
	Chicken with skin	0.47309	
	Chips	0.46882	
	Cold meat	0.46529	
	Red meat	0.43206	
	Flame-grilled fast-food chicken	0.42456	
	Hot dogs	0.40035	
	Sausages	0.39612	
	Fizzy soft drinks	0.38538	
	Fruit juice	0.35977	
	Hamburgers	0.31074	
	Pizza	0.30266	
	Rusks	0.30247	
	Cooking oil	−0.33744	
“Spreads and fast foods”	Dripping	0.76641	11.12%
	Salad dressing low fat	0.76498	
	Fat Holsum	0.74403	
	Schnitzels	0.70762	
	Skimmed	0.6791	
	Chocolate spread	0.60188	
	Bunny chow	0.58556	
	Venison	0.55576	
	Fizzy diet soft drinks	0.51667	
	Fish steamed	0.50548	
	Whole wheat	0.49309	
	Dried fruit	0.47822	
	Red meat fat removed	0.47779	
	Cookies	0.45296	
	Fried fast food chicken	0.4281	
	Pasta	0.42541	
	Organ meat	0.40937	
	Nuts and peanuts	0.36348	
	Pizza	0.34863	
	Pies and sausage rolls	0.34244	
	Vetkoek	0.33541	
	Rusks	0.32428	
	Hot dogs	0.31508	
	Butter	0.3194	
	Fizzy soft drinks	−0.31292	
“Butter, junk foods and juices”	Butter	0.623	10.45%
	Sweets	0.60625	
	Muffins	0.59219	
	Chips	0.56772	
	Mixed salad	0.56106	
	Fruit juice	0.5389	
	Fresh fruit	0.53471	
	Fizzy soft drinks	0.42281	
	Vetkoek	0.39649	
	Coffee creamer	0.38703	
	Cooking oil	0.38482	
	Hamburgers	0.36097	
	Cooked vegetables	0.35781	
	Cereals Rice Crispies	0.31854	
	Soft margarine	−0.77394	
“Protein-rich foods”	Fried fish	0.67237	6.35%
	Fish tinned	0.54825	
	Fried fish in fat	0.52289	
	Eggs cooked or poached	0.46767	
	White brown bread	0.43925	
	Potato chips	0.4079	
	Chicken with skin	0.40252	
	Chicken without skin	−0.34525	
	Red meat fat removed	−0.40646	
“Starch foods”	Potato	0.66418	4.56%
	Breakfast cereals	0.52251	
	Potato with fat	0.47055	
	Legumes	0.45527	
	Full cream	0.40847	
	Cooked vegetables	0.36178	
	Cheese sauce	0.35377	
	Pasta	0.32992	
	Jam	0.32221	
“Nuts and rice foods”	Peanut butter	0.55276	3.63%
	Nuts and peanuts	0.39124	
	Rice mealie rice	0.34955	
“Vegetable-rich foods”	Vegetables	0.61485	3.13%
	Organ meat	0.40243	
	Butter	−0.34338	
“Alcoholic drinks”	Shooters	0.80795	2.67%
	Cocktails	0.79207	

*Factor loadings ≥3.0 or ≤−3.0. Food groups are sorted by the size of loading coefficients.

### Data exploration

**Figure 2:**
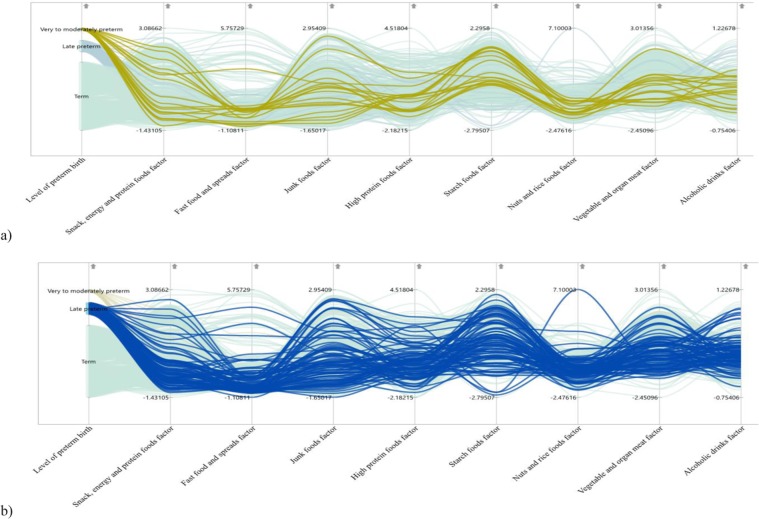
Parallel coordinates plot for trends of latent dietary patterns by levels (**a**) very/moderately (**b**) late preterm birth.

Exploration of the data was performed using parallel coordinate plots (PCP), in order to examine trends of dietary patterns across different severity levels of preterm birth. The PCP revealed an association between the higher-order severity levels 2 and 3 of preterm birth outcomes, with lower consumption of ‘nuts and rice foods’ dietary patterns (Fig. 2). Except for some outliers, similar PCP trends were observed for ‘spreads and fast foods’ dietary patterns. The PCP further displayed that the higher-order severity levels 2 and 3 of preterm birth, had a high range of variation from low to high on ‘butter, junk foods, and juices’ and ‘energy foods and snacks’ dietary patterns, with high clustering of late preterm birth at lower scores (Fig. 2). This supports the use of a model (such as the partial proportional odds model) that considers the differential effect of dietary patterns across the severity levels of preterm birth.

### Statistical analysis

Gestational age was categorized into three levels, based on the severity levels of preterm birth. The outcome variable of interest (severity level of preterm birth) was measured on three ordinal levels of term (38–42 weeks), late preterm (34–37 weeks) and very (<32 weeks) or moderately preterm (32–33 weeks) birth. These sub-categories of preterm birth were coded to represent severity levels: term birth = 1, late preterm birth = 2, very or moderately preterm birth (most severe) = 3. The level k = 1 defines the lowest severity level (term birth). Therefore, for the i^th^ woman in the study, the response variable, the severity level of preterm birth (Y_i_), i = 1… n, is defined as follows

$${Y}_{i}=\{\begin{array}{c}1:{\rm{if}}\,{\rm{gestational}}\,{\rm{age}}\,{\rm{is}}\,{\rm{between}}\,38\,{\rm{and}}\,42\,{\rm{weeks}}\,\\ 2:{\rm{if}}\,{\rm{gestational}}\,{\rm{age}}\,{\rm{is}}\,{\rm{between}}\,34\,{\rm{and}}\,37\,{\rm{weeks}}\\ 3:\,{\rm{if}}\,{\rm{gestational}}\,{\rm{age}}\,{\rm{is}}\,{\rm{between}}\,23\,{\rm{and}}\,34\,{\rm{weeks}}\end{array}$$

A proportional odds model is one in which the response variable has more than two ordinal categories, with the assumption of odds being the same across the categories. The partial proportional odds model is suitable in modeling severity levels of preterm birth due to the flexibility in the procedure, as it is capable of relaxing the proportional odds assumption in the ordered logit model, by allowing the variability of the regression parameter *β*, across severity levels of preterm birth, while maintaining its ordinal nature. It estimates the cumulative probabilities of being at or below, specific severity levels of preterm birth, given a set of independent variables^33–35,40^. The partial proportional odds model can be given as

1 $$logit\left[\frac{P(Y > j/{x}_{1}\ldots {x}_{p})}{P(Y\le j/{x}_{1}\ldots {x}_{p})}\right]={\alpha }_{j}+{X}_{i}{\beta }_{j}\,j=1,2,\ldots ,k-1$$

where Y is the ordinal response severity levels of preterm birth, **x** is the vector of observed explanatory variables: maternal and infant characteristics, including maternal dietary patterns, maternal age, maternal education, employment, maternal annual income, family size, gravida, and infant gender, ***β*** is the vector of estimable parameters and **α** is the unknown threshold, or intercept parameters. Consequently, the model in Eq. 1 is the partial proportional odds model.

The proportional odds, in particular, the probability of an observation being at, or above, a specific severity level of preterm birth, was conditional on maternal dietary practices and socio-demographic characteristics observed in the study. In fitting a partial proportional odds model, it is imperative to verify the homogeneity of the proportional odds ratios across all ordinal levels, using a global test of non-proportionality^41^. The predictors that met the proportional odds assumption, dietary patterns (‘energy foods and snacks’, ‘spreads and fast foods’, ‘butter, junk foods, and juices’, ‘protein-rich foods’, ‘vegetable-rich foods and alcoholic drinks’) and covariates (maternal employment, maternal age, maternal education and infant gender), have the same coefficient values in two of the cumulative logits, the higher severity levels (levels 2 or 3) versus the lowest level (level 1) and the most severe level (level 3) versus the lower severity levels (levels 1 or 2). We considered p-values below 0.05 to be statistically significant. SAS 9.4 was used for the analysis of the data.

### Ethics approval and consent to participate

Written, informed consent was obtained from the mother for the children and provided their own consent. The study was approved by the Biomedical Research Ethics Committee of the University of KwaZulu-Natal.

## Results

The proportion of preterm birth in the MACE cohort was 17%, with 14% late preterm birth and about 3% very to moderately preterm births. As for the profile of the mothers, the majority were between 20 and 29 years of age (60.4%), multigravida (57.1%), high school graduates (79.4%), unemployed (81.5%) and had no personal income (45.3%). Male babies predominated (52.1%).

### Model fitting and comparison

The Score test indicated that the proportional odds assumption is not reasonable, suggesting that separate parameters are needed across the cumulative logits for at least one predictor (p = 0.006). Thus, we conducted model comparison among the three ordinal logistic regression models, with likelihood ratio tests. The proportional odds model was rejected in favor of both the non-proportional odds model (p = 0.001) and the partial proportional odds model (p = 0.0001), while the partial proportional odds model fits as well as the non-proportional odds model (p = 0.305). The Akaike Information Criterion (AIC) and Bayesian Information Criterion (BIC) values were used to compare the performance of the non-proportional and partial proportional odds models. The partial proportional odds model had the lowest AIC and BIC values, compared to the non-proportional odds model (Table 2). This suggested that the partial proportional odds model outperformed the non-proportional models and could be considered as a viable method for modeling the severity level of preterm birth.

**Table 2:** Model comparison values based on likelihood ratio tests, Akaike Information Criterion (AIC) and Bayesian Information Criterion (BIC), for three proportional odds models.

Test	*χ*^2^	DF	p
Non- proportional odds model vs. proportional odds model	41.17	18	0.001
partial proportional odds model vs. proportional odds model	27.25	6	0.0001
Non-proportional odds model vs. partial proportional odds model	13.92	12	0.305
Non- proportional odds model	AIC = 673.93	BIC = 844.29	
partial proportional odds model	AIC = 663.85	BIC = 780.41	

The proportional odds assumption was violated for ‘starch foods’, ‘nuts and rice foods’ dietary patterns and maternal income, family size and gravida, among socio-demographic factors. The dietary patterns ‘energy foods and snacks’, ‘spreads and fast foods’, ‘butter, junk foods, and juices’ and ‘protein-rich foods’, ‘vegetable-rich foods’, ‘alcoholic drinks’ and the adjusted socio-demographic variables maternal age, education, employment, and infant gender, all satisfied the proportional odds assumption.

The likelihood ratio chi-square test for the partial proportional odds model was significant with χ^2^ (36) = 57.42, p < 0.0001, indicating that the full model with predictors, provided a better fit than the null model, with parameters significantly being non zero (Table 3). The partial proportional odds model revealed that the two dietary patterns, ‘starch foods’ and ‘nuts and rice foods’, as well as family size, showed a significant differential effect across severity levels of preterm birth. These dietary patterns showed a statistically significant association with highest severity preterm birth, against lower severity levels 1 or 2 of preterm birth outcomes. A mother who consumed ‘starch foods’ had the greatest risk of having the most severe (level 3) preterm birth category instead of lower severity levels 1 or 2 (Adjusted Odds Ratio (AOR) = 2.09, 95% CI = (1.16, 3.80), p = 0.014). However, the risk of having the most severe preterm birth, as compared to the other categories, decreased by 75% (AOR = 0.25, 95% CI = (0.099, 0.621), p = 0.003), for each unit increase in intake of the ‘nuts and rice foods’ diet (Table 3).

**Table 3:** Partial proportional odds model for severity levels of preterm birth.

Factors	Level 2, Level 3 vs Level 1				Level 3 vs level 1, level 2			
	*β*_*1*_	SE	95%CI	AOR	*β*_*2*_	SE	95%CI	AOR
Intercept	−2.5363*	0.5234			−7.2263*	1.2210		
^†^Energy foods and snacks	−0.2272	0.1633	(0.579, 1.097)	0.797	−0.2272	0.1633	(0.579, 1.097)	0.797
^†^Spreads and fast foods	0.1736	0.1488	(0.889, 1.592)	1.190	0.1736	0.1488	(0.889, 1.592)	1.190
^†^Butter, junk foods and juice	−0.0121	0.1593	(0.723, 1.350)	0.988	−0.0121	0.1593	(0.723, 1.350)	0.988
^†^Protein rich foods	−0.0307	0.1458	(0.729, 1.291)	0.970	−0.0307	0.1458	(0.729, 1.291)	0.970
Starch foods	−0.1611	0.1255	(0.666, 1.088)	0.851	0.7370*	0.3010	(1.158, 3.769)	2.090
Nuts and rice foods	−0.0217	0.1354	(0.751, 1.276)	0.979	−1.3956*	0.4688	(0.099, 0.621)	0.248
^†^vegetable rich foods	−0.3148*	0.1619	(0.531, 0.981)	0.730	−0.3148*	0.1619	(0.531, 0.981)	0.730
^†^Alcoholic drinks	1.2543*	0.5185	(1.269, 9.684)	3.505	1.2543*	0.5185	(1.269, 9.684)	3.505
^**†**^**Maternal age (Prime of fertility(20–29 years)**^**a**^**)**								
Teen age (15–19 years)	0.1676	0.3304	(0.619, 2.259)	1.182	0.1676	0.3304	(0.619, 2.259)	1.182
Age of 30 years and above)	0.5664*	0.2782	(1.021, 3.039)	1.762	0.5664*	0.2782	(1.021, 3.039)	1.762
^**†**^**Maternal education (College or university**^**a**^**)**								
Primary or less	0.9499	0.6627	(0.705, 9.476)	2.585	0.9499	0.6627	(0.705, 9.476)	2.585
High school	0.2117	0.3303	(0.647, 2.361)	1.236	0.2117	0.3303	(0.647, 2.361)	1.236
^**†**^**Employment (Unemployed**^**a**^**)**								
Employed	0.2574	0.3052	(0.711, 2.353)	1.294	0.2574	0.3052	(0.711, 2.353)	1.294
**Maternal annual income ($2000 and above**^**a**^**)**								
No personal income	0.2106	0.3906	(0.574, 2.654)	1.234	0.4944	1.1064	(0.187,14.339)	1.640
Less than $2000	0.1745	0.3984	(0.545, 2.600)	1.191	1.6579	1.0444	(0.634, 40.644)	5.248
Family size	0.0573	0.0709	(0.922, 1.217)	1.059	0.4055*	0.1342	(1.153, 1.951)	1.500
**Gravida (Multigravida**^**a**^**)**								
Primagravida	−0.0540	0.2698	(0.558, 1.608)	0.947	0.1038	0.5447	(0.381, 3.227)	1.109
^**†**^**Infant gender (Male**^**a**^**)**								
**Female**	0.0715	0.2209	(0.697, 1.656)	1.074	0.0715	0.2209	(0.697, 1.656)	1.074
Score test for proportional odds assumption	*χ*^2^ = 36.187 Df = 18 p-value = 0.006							
Goodness of fit (likelihood ratio)	*χ*^2^ = 57.4216 Df = 24 p-value < 0.0001							

*Significant at 0.05 level. ^a^Reference category ^†^proportional odds assumption holds and have the same coefficient values in the two cumulative logits.

The consumption of ‘vegetable-rich foods’ and ‘alcoholic drinks’ latent dietary patterns had a significant effect that did not vary across severity levels of preterm birth. The odds of having higher severity levels 2 and 3 preterm birth compared to level 1, decreased by 27% for each unit increase of ‘vegetable-rich foods’ (AOR = 0.73, (95% CI = (0.531, 0.981), p = 0.036) (Table 3). There was a 3.5 fold (95% CI = (1.269, 9.684), p = 0.015) increased risk for preterm birth at a higher severity level, i.e. level 2 or 3, compared to severity level preterm birth 1, for one unit increased consumption of alcoholic drinks (Table 3). Furthermore, among the adjusted socio-demographic variables, the risk of having the most severe preterm birth outcome against lower levels was increased for every one additional person in the family (AOR = 1.50, 95% CI = (1.153, 1.951), p = 0.002), and being in the age group of 30 years and above (AOR = 1.76, 95% CI = (1.021, 3.069), p = 0.041) (Table 3).

## Discussions

The differential effect of maternal dietary patterns across different severity levels of preterm birth was examined using the partial proportional odds ordinal model. The result showed a significant protective effect of ‘vegetable-rich foods’, as well as ‘nuts and rice foods’, for experiencing the most severe preterm birth category. Previous prospective cohort studies^42,43^ found that the Mediterranean-style dietary pattern, characterized by high consumption of fruits and vegetables during pregnancy, was associated with a decrease in the risk of late preterm birth. However, we found that an increase in consumption of a ‘vegetable-rich foods’ dietary pattern, was related to a decreased risk of preterm birth at both very/moderate and late preterm birth. This may possibly be due to the high loading of vegetables and negative loading of butter, implying that a maternal diet, with frequent consumption of vegetables, could contribute to a lowered odds of preterm birth. This is similar to the finding of a study in Norway, which found evidence that increasing scores for the “prudent” dietary patterns, characterized by diets which are rich in vegetables and fruits, was found to be associated with a lowered risk of preterm birth as a whole^23^.

Our findings are also consistent with the outcomes of a study of dietary pattern and its association with preterm birth in Singapore, that showed a dietary pattern high in vegetables, fruits, and white rice, is associated with a lower risk of preterm birth^44^. Our findings are further consistent with a study in China, which showed that maternal diet with frequent consumption of vegetables, might contribute significantly to lowering odds of experiencing a preterm birth outcome^30^. A study in Singapore found that the consumption of the ‘vegetable, fruit and rice’ pattern, which includes ‘nut and rice foods’, was found to be associated with a reduced risk of preterm birth outcome^45^. Likewise, our investigation of the consumption of ‘nuts and rice foods’ varied between different severity levels of preterm birth and also had evidence of lower risk of having very/moderately preterm birth outcome, as compared to late preterm or term birth outcome.

A Norwegian mother and child cohort study^23^, found that an increase in scores of the “traditional” (potatoes, fish, boiled vegetables) dietary pattern, was associated with a lower risk of preterm birth. However, our study showed that women who follow ‘starch foods’ dietary pattern (characterized by high consumption of potatoes, breakfast cereals, potatoes with fat, legumes, cooked vegetables), is associated with the most severe level of preterm birth. Here, the effect of ‘starch foods’ dietary pattern, varied across the different severity levels of preterm birth. This is due to the use of the partial proportional odds model, which considered the severity level of preterm birth, on top of other studies that use the multinomial logistic regression model. This may also be attributed to the diets having a high glycemic index, such as potatoes and potatoes fried with fat. These have a high loading on the starch foods dietary pattern by increasing blood glucose levels, similar to the study focusing on pregnant women with diabetes, who deliver preterm babies^46,47^. An increase in consumption of alcoholic drinks was associated with a higher risk of preterm birth, this association being similar across all severity levels of preterm births. Moreover, the study indicated that older age mothers had a significantly higher risk of preterm birth across all severity levels. This is consistent with other studies that showed that the risk of having a preterm baby was higher for older mothers, and further that this risk increased, with an increase in severity level of preterm birth^39,48^.

The strength of this study lies in the use of a more parsimonious method, the partial proportional odds model, that allows the predictors that meet the proportional odds assumption to take the same coefficient for all severity levels of preterm birth, and other predictors to vary across the severity levels of preterm birth, thereby ensuring that there is no potential loss in accuracy of prediction.

Ordinal models assume the proportional odds assumption across severity levels of preterm births, compared to multinomial logit models, which completely ignore the sequential order of preterm births. The assumption of proportional odds model, however, was not supported by the data for all the factors. The partial proportional odds model bridges the gap between the proportional odds and the multinomial logit models^41^. As a result, we used the partial proportional odds model, which accommodates the variables that failed to satisfy the proportional odds model assumption. Unlike previous studies, which were based on the effect of consumption of single food items, a data-driven exploratory factor analysis was used to extract dietary patterns that facilitate understanding of a variety of food consumption habits of the cohort. The limitation of the study is that the data is only adjusted for socio-demographic factors. It is possible that our findings could be biased due to unmeasured confounding variables.

## Conclusions

This study found that the ‘vegetable-rich foods’ and ‘nuts and rice foods’ dietary patterns were associated with a reduced likelihood of preterm birth. The dietary patterns such as ‘starch foods’ and ‘alcoholic drinks’ and older age were factors that were associated with an increased likelihood of preterm birth. The study further showed ‘starch foods’ and ‘nuts and rice foods’ dietary patterns, as well as family size, had a differential effect at different severity levels of preterm birth. The use of partial proportional odds model allowed for modeling the effect of dietary patterns on the severity of preterm birth, allowing for flexibility over the assumption of homogeneity of threshold-specific covariate effects and may consequently allow for a more proper application of models for ordered responses, than is the case for the standard proportional odds methods.

## Data Availability

The data that support the findings of this study are available from the MACE study but restrictions apply to the availability of these data, which were used under license for the current study, hence are not publicly available. Data are however available from the authors upon reasonable request and with permission from the MACE study.
